# Retrospective comparison between breast cancer tissue- and blood-based next-generation sequencing results in detection of *PIK3CA*, *AKT1*, and *PTEN* alterations

**DOI:** 10.1186/s13058-025-02055-0

**Published:** 2025-07-01

**Authors:** Moumita Chaki, Mona Benrashid, Subir Puri, Smruthy Sivakumar, Ethan S. Sokol, Josefa M. Briceno, Neil Vasan

**Affiliations:** 1 Breast Cancer, US Medical Affairs, AstraZeneca, Gaithersburg, MD USA; 2 Computational Discovery, Foundation Medicine Inc, Cambridge, MA USA; 3 NYU Langone Health, New York, NY USA

**Keywords:** AKT inhibitor, Capivasertib, Targeted therapy, Breast cancer, Next-generation sequencing, Circulating tumor DNA, HR-positive/HER2-negative

## Abstract

**Background:**

Based on the CAPItello-291 phase III trial results, capivasertib in combination with fulvestrant has been approved for patients with hormone receptor-positive/human epidermal growth factor receptor 2-negative advanced breast cancer harboring one or more *PIK3CA*, *AKT1*, and/or *PTEN* alterations. Given the growing interest in circulating tumor DNA (ctDNA) next-generation sequencing (NGS) to detect *PIK3CA/AKT1/PTEN* alterations, we retrospectively compared blood-based FoundationOne®Liquid CDx versus tumor tissue-based FoundationOne®CDx real-world data from patients with various breast cancer subtypes.

**Methods:**

We utilized a database of patients profiled with FoundationOne®CDx and/or FoundationOne®Liquid CDx during routine clinical care. Analytical comparison of all pathogenic alterations in *PIK3CA*, *AKT1*, *AKT2*, *AKT3,* and *PTEN,* including alterations defined in the CAPItello-291 protocol (CAPItello-defined alterations), was performed in paired data from 289 patients with both tissue and liquid biopsies sampled within 90 days of each other.

**Results:**

Overall positive percent agreement (PPA) for short variants across ctDNA tumor fraction (TF) subgroups in paired biopsy samples was: ctDNA TF ≥ 10%: *PIK3CA*, 93.9%; *AKT1*, 100%; *PTEN*, 100%; ctDNA TF 1%-10%: *PIK3CA*, 96.3%; *AKT1*, 100%; *PTEN*, 100%; ctDNA TF < 1%: *PIK3CA*, 34.7%; *AKT1*, 50.0%; *PTEN*, 37.5%. PPA for CAPItello-defined alterations was: ctDNA TF ≥ 10%: 92.5%; ctDNA TF 1%-10%: 97.1%; ctDNA TF < 1%: 33.9%. For *PTEN* homozygous deletions, PPA was 50.0% in cases with ctDNA TF ≥ 10%. Overall PPA for *AKT2* and *AKT3* copy number variations (CNVs) was 66.7% and 0%, respectively.

**Conclusions:**

Blood-based NGS could offer a minimally invasive option to identify clinically relevant *PIK3CA*/*AKT1*/*PTEN* short variants in cases with ctDNA TF ≥ 1%. Confirmatory tissue-based NGS should be performed when blood-based NGS results are negative, especially when ctDNA TF is < 1% and for enhanced detection of CNVs in general.

**Supplementary Information:**

The online version contains supplementary material available at 10.1186/s13058-025-02055-0.

## Introduction

The phosphatidylinositol-3-kinase (PI3K)/Akt serine/threonine kinase (AKT) pathway is frequently hyperactivated in cancers, promoting uncontrolled tumor growth, angiogenesis, and metastasis [1]. Activating mutations in *PIK3CA* (PI3K catalytic subunit alpha) and *AKT1* (Akt serine/threonine kinase 1), and inactivating alterations involving *PTEN* (phosphatase and tensin homolog) linked to pathway hyperactivation, occur in about half of hormone receptor (HR)-positive/human epidermal growth factor receptor 2 (HER2)-negative breast cancers [2–5].

In the randomized phase III, CAPItello-291 trial, the efficacy and safety of the addition of capivasertib (an oral inhibitor of all AKT isoforms) to fulvestrant was examined in patients with HR-positive/HER2-negative advanced breast cancer who had a relapse or disease progression during or after treatment with an aromatase inhibitor [6]. Capivasertib–fulvestrant demonstrated statistically significant and clinically meaningful improvement compared with placebo–fulvestrant in the dual primary endpoints of progression-free survival in patients with *PIK3CA/AKT1/PTEN*-altered tumors (hazard ratio, 0.50; 95% CI, 0.38 to 0.65, *P* < 0.001), and in the overall population (hazard ratio, 0.60; 95% CI, 0.51 to 0.71, *P* < 0.001) [6]. Testing for *PIK3CA/AKT1/PTEN* alterations was carried out using FoundationOne®CDx, a tumor tissue-based next-generation sequencing (NGS) test, for all patients except those enrolled in China, where OncoScreen Plus® was used instead [6]. Based on the above results, a first-in-class approval was granted by the US Food and Drug Administration (FDA) for capivasertib, in combination with fulvestrant, in patients with tumors harboring one or more *PIK3CA*, *AKT1*, and/or *PTEN* alterations [7] detected by the FDA-approved companion diagnostic test, FoundationOne®CDx [8]. Subsequently, capivasertib–fulvestrant was added to clinical guidelines as a treatment option for certain patients [9, 10]. Of note, the PI3K inhibitors alpelisib, in combination with fulvestrant [9, 10], and inavolisib, in combination with palbociclib and fulvestrant [10], are indicated for certain patients with HR-positive/HER2-negative advanced breast cancer with tumors harboring *PIK3CA*-activating mutations. The FDA-approved companion diagnostic tests are FoundationOne®CDx, FoundationOne®Liquid CDx, or the *therascreen*® PIK3CA RGQ PCR Kit for alpelisib, and FoundationOne®Liquid CDx for inavolisib [8].

NGS-based testing has been widely adopted in oncology clinical practice for the identification of tumor genomic alterations. In patients with advanced breast cancer, guidelines recommend the use of genomic profiling/NGS where there is clinical value in guiding treatment decision making [10, 11]. Multigene NGS panels enable detection of single nucleotide variants (SNVs), insertions/deletions, copy number variants (CNVs), and structural rearrangements, and offer a rapid, cost-effective alternative to whole-exome sequencing and whole-genome sequencing [12]. With robust and reproducible workflows in place, tumor tissue-based genomic profiling is commonly utilized for biomarker detection to guide treatment decisions [13]. Compared with tumor tissue-based testing, blood-based testing is minimally invasive, has a faster turnaround time [14], and can provide a real-time snapshot of the genomic landscape across multiple tumor sites [15, 16]. Furthermore, in cases where tumor tissue is not available in the metastatic setting (e.g. when obtaining tumor tissue from the metastatic site [such as bone] is challenging [17, 18]; when there are difficulties tracing an archival tumor sample; or when samples do not have sufficient tumor cell content), blood-based testing can provide crucial information. However, blood-based testing is associated with technical limitations, such as low or absent circulating tumor DNA (ctDNA) resulting in low tumor fraction (TF; representing the fraction of circulating cell-free DNA originating from a tumor [19]) in some patients [13, 20], and reduced sensitivity in the detection of CNVs and rearrangements [21, 22], such as *PTEN* homozygous deletions and rearrangements [23, 24]. Currently, guidelines recommend reflex to confirmatory tissue-based testing for detection of certain biomarkers if no alterations are detected using blood-based NGS [10].

Given the growing utility of ctDNA and the increased interest in blood-based tests to detect *PIK3CA/AKT1/PTEN* alterations, we performed a retrospective analytical comparison of data from patients tested with blood-based FoundationOne®Liquid CDx NGS versus tissue-based FoundationOne®CDx NGS during routine clinical care. We assessed detection of: 1) all pathogenic alterations within/involving *PIK3CA*, *AKT1*, *AKT2*, *AKT3*, and *PTEN* detected by FoundationOne®CDx and FoundationOne®LiquidCDx; and 2) *PIK3CA/AKT1/PTEN* alterations defined in the protocol of CAPItello-291 (i.e., CAPItello-defined alterations). Of note, unlike in CAPItello-291, the results reported here are not specific to patients with HR-positive/HER2-negative advanced breast cancer, as our cohort included patients with various breast cancer subtypes, regardless of HR/HER2 status.

## Methods

### Comprehensive Genomic Profiling (CGP)

We conducted a retrospective analysis of data from patients with various breast cancer subtypes (not limited to HR-positive/HER2-negative) who received tumor tissue-based CGP and/or blood-based CGP during routine clinical care at Foundation Medicine. Tissue-based CGP was performed using FoundationOne®CDx (Foundation Medicine, Inc., Boston, Massachusetts, United States) on formalin-fixed, paraffin-embedded tissue biopsy sections [25]. Tissue samples were from primary or metastatic disease and were collected at original resection or recurrence. Blood-based CGP utilized the blood-based FoundationOne®Liquid CDx assay (Foundation Medicine, Inc., Boston, Massachusetts, United States) [26]. Whole blood was freshly collected in tubes provided with the FoundationOne®Liquid CDx Specimen Collection and Shipping Kit, mixed gently by inversion, and shipped at room temperature, preferably on the same day of collection. Details on the sequencing and analytical validation of the two assays have been described previously [25, 26]. Hybrid capture was carried out for 324 cancer-associated genes to detect short variants (SVs; typically below 200 bp in length, including SNVs and insertions/deletions), CNVs (gene amplifications and homozygous deletions), and genomic rearrangements. For blood-based CGP, homozygous deletions are reported for *PTEN*, *BRCA1*, and *BRCA2*. Approval for this study, including a waiver of informed consent and a Health Insurance Portability and Accountability Act (HIPAA) Waiver of Authorization, was obtained from the Western Institutional Review Board (protocol number 20152817).

### Estimation of ctDNA tumor fraction in liquid biopsies

For blood samples with significant aneuploidy, the copy number modeling-based purity assessment was used to estimate ctDNA TF [27]. If significant aneuploidy was not detected, variant allele frequencies (VAFs) of the detected somatic SVs and rearrangements were used to estimate ctDNA TF. Somatic status for SVs and rearrangements was determined either by presence in a whitelist of specific variants and categories of variants that are highly biased toward being somatic (and not due to clonal hematopoiesis), or by evidence of a statistically significant difference in quantitative metrics from fragments harboring the mutated allele. While not all true somatic variants for a given sample are identified by these approaches, a substantial majority of samples have ≥ 1 eligible variant for estimation. The ctDNA TF estimate may then include the highest VAF for an SV or rearrangement deemed to be somatic.

### Comparison of tissue- and blood-based profiling in breast cancer

The prevalence of all pathogenic/likely pathogenic alterations (denoted as “pathogenic alterations” throughout the paper) in *PIK3CA*, *AKT1*, *AKT2*, *AKT3*, and *PTEN*, and of CAPItello-defined alterations, was compared between the overall tissue and liquid biopsy cohorts. A list of CAPItello-defined alterations is given in Table S1. Genomic alterations in *PIK3CA*, *AKT1*, *AKT2*, *AKT3*, and *PTEN* were designated as pathogenic, as described previously [28], using multiple annotations such as: 1) presence in the COSMIC database, 2) additional knowledge about the gene affected, such as truncations and deletions in known tumor suppressor genes, or 3) characterization as pathogenic in the scientific literature. All other uncharacterized alterations were denoted variants of unknown significance. Concordance for all pathogenic alterations in *PIK3CA*, *AKT1*, *AKT2*, *AKT3*, and *PTEN*, including CAPItello-defined alterations, was evaluated using paired data from patients with both tissue- and blood-based CGP. Analysis was performed separately for SVs and CNVs. Rearrangement-based analysis was not performed, as there was only one case with a *PTEN* rearrangement across all paired biopsies. Analysis was limited to patient-paired tissue and liquid biopsies collected within 90 days of each other, to ensure contemporaneousness of sampling in relation to disease course; tissue biopsy collection could precede liquid biopsy collection, or vice versa. Collection refers to the date of sample collection (i.e., if a historical sample was used, the collection date would be the biopsy date, not the date the archival tissue was accessed). Positive (PPA) and negative percent agreement (NPA) for results from blood- versus tissue-based CGP were calculated as follows:

$$\text{PPA}= \frac{\text{shared positive}}{\text{shared positive }+\text{ tissue-only positive}}$$

where shared positive: samples (*n*) with both tissue- and blood-based CGP positive results; tissue-only positive: samples (*n*) with positive results in tissue-based CGP only

$$\text{NPA}= \frac{\text{shared negative}}{\text{shared negative }+\text{ liquid-only positive}}$$

where shared negative: samples (*n*) with both tissue- and blood-based CGP negative results, liquid-only positive: samples (*n*) with positive results in blood-based CGP only.

PPA and NPA were evaluated based on the ctDNA TF for the liquid biopsies (ctDNA TF < 1%, 1%-10%, and ≥ 10%) and on the collection interval between paired biopsies (< 30 days and 30–90 days).

### Patterns of co-occurring pathogenic alterations

Using the overall liquid biopsy cohort of patients with various breast cancer types, prevalence of co-occurring pathogenic alterations in other genes was examined in samples with CAPItello-defined alterations and, separately, with any *PIK3CA*, *AKT1*, and *PTEN* pathogenic alterations. A detailed breakdown of the prevalence of individual estrogen receptor 1 (*ESR1*) alterations was performed. We also present the combined prevalence of *ESR1* driver mutations, previously described, [29] comprising E380Q, S463P, P535H, L536R, L536Q, L536H, L536P, Y537S, Y537N, Y537C, and D538G. Additionally, we identified samples with *ESR1* alterations in the overall liquid biopsy cohort and determined the prevalence of CAPItello-defined alterations within this *ESR1* alteration-positive cohort.

### Statistical analysis

A Fisher’s exact test was used to compare the prevalence of gene alterations between the tissue- and blood-based assays. Two-sided *P* values were calculated, followed by multiplicity adjustment using the Benjamini–Hochberg false discovery rate (FDR) method; an FDR-adjusted *P* value ≤ 0.05 was considered statistically significant. Statistics, computation, and plotting were done using Python 3.9.12 (Python Software Foundation, Delaware, United States), R 4.3.1 (R Foundation for Statistical Computing, Vienna, Austria), and Microsoft Excel 365 v2402 (Microsoft Corporation, Redmond, Washington, United States).

## Results

### Study population

The analysis included 35,730 patients who received tumor tissue-based CGP (tissue biopsy cohort) and 7,056 patients who received blood-based CGP (liquid biopsy cohort) from January 2017 to June 2023 (Fig S1). Within the liquid biopsy cohort, 3,344 patients (47.4%) had samples with ctDNA TF ≥ 1% (liquid biopsy cohort ctDNA TF ≥ 1%). The median (interquartile range) age was 61 (51–69) years in the overall tissue biopsy cohort and 65 (56–73) years in the overall liquid biopsy cohort. Most patients had metastatic (stage IV) breast cancer (Table 1). Paired data from both tissue- and blood-based CGP were available for 289 patients. Characteristics for the liquid biopsy cohort with ctDNA TF ≥ 1% (Table S2) and for the patients with paired CGP data (Table S3) were generally similar to those for the overall cohorts. Information on HR/HER2 status was not routinely available; therefore, the study population included patients with breast cancer with variable HR/HER2 status, including HR-positive breast cancer and triple-negative breast cancer (TNBC).

**Table 1:** Patient characteristics

Characteristic	Tissue biopsy cohort (*N* = 35,730)	Liquid biopsy cohort (*N* = 7,056)
Age (years), median (interquartile range)	61 (51–69)	65 (56–73)
Sex, *n* (%)		
Male	413 (1.2)	84 (1.2)
Female	35,311 (98.8)	6,971 (98.8)
Unknown	6 (< 0.1)	1 (< 0.1)
Tumor type, *n* (%)		
Breast/breast carcinoma (not otherwise specified)	21,331 (59.7)	5,588 (79.2)
Breast invasive ductal carcinoma	11,640 (32.6)	1,147 (16.3)
Breast invasive lobular carcinoma	2,213 (6.2)	299 (4.2)
Breast metaplastic carcinoma	385 (1.1)	7 (0.1)
Other breast^a^	161 (0.5)	15 (0.3)
Tumor stage, *n* (%)		
I	893 (2.5)	183 (2.6)
II	1,936 (5.4)	320 (4.5)
III	2,604 (7.3)	337 (4.8)
IV	22,268 (62.3)	4,709 (66.7)
Unknown	8,029 (22.5)	1,507 (21.4)

Please note that information on hormone receptor and human epidermal growth factor receptor 2 status (including hormone receptor-positive and triple-negative breast cancers) was not routinely available. ^a^Other breast: breast phyllodes tumor, breast mucinous carcinoma, breast papillary carcinoma, breast ductal carcinoma in situ, breast inflammatory carcinoma, breast carcinosarcoma, breast lobular carcinoma in situ, breast myoepithelial carcinoma

### Prevalence of pathogenic alterations in *PIK3CA*, *AKT1*, *AKT2*, *AKT3*, and *PTEN* in the tissue and liquid biopsy cohorts

**Fig. 1:**
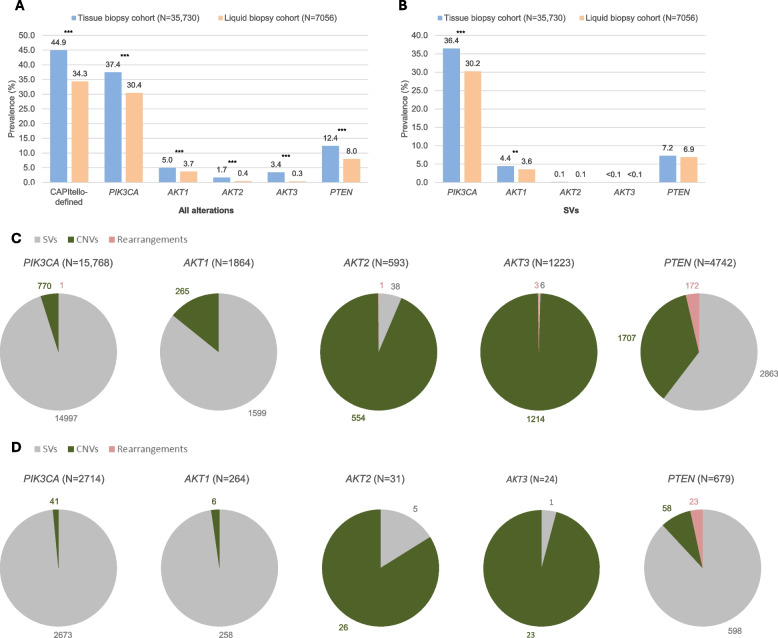
Prevalence of (**A**) all pathogenic alterations and (**B**) pathogenic SVs in the tissue and liquid biopsy cohorts, and number of alterations (SVs, CNVs, and rearrangements) detected in (**C**) the tissue biopsy cohort and (**D**) the liquid biopsy cohort. **P < .01; ***P < .001. Please note that information on hormone receptor and human epidermal growth factor receptor 2 status (including hormone receptor-positive and triple-negative breast cancers) was not routinely available. (**A**, **B**) *N* = total number of samples; (**C**, **D**) *N* = total number of detected alterations per gene. *AKT1*, Akt serine/threonine kinase 1; *AKT2*, Akt serine/threonine kinase 2; *AKT3*, Akt serine/threonine kinase 3; CNV, copy number variant; *PIK3CA*, phosphatidylinositol-3-kinase catalytic subunit alpha; *PTEN*, phosphatase and tensin homolog; SV, short variant

Prevalence of all pathogenic alterations in *PIK3CA*, *AKT1, AKT2, AKT3,* and *PTEN* was significantly higher in the tissue versus the non-paired liquid biopsy cohort (37.4% versus 30.4%, 5.0% versus 3.7%, 1.7% versus 0.4%, 3.4% versus 0.3%, and 12.4% versus 8.0%, respectively; Fig. 1A). When assessing prevalence of SVs in the tissue versus liquid biopsy cohorts, statistically significant differences were observed for *PIK3CA* and *AKT1* alterations (36.4% versus 30.2%, and 4.4% versus 3.6%, respectively; Fig. 1B). Pathogenic alterations detected in *PIK3CA* and *AKT1* were predominantly SVs, while alterations involving *PTEN* were also most commonly SVs, albeit with a higher prevalence of CNVs, underlying the importance of reliable SV detection through tissue- and blood-based testing. *AKT2* and *AKT3* alterations were predominantly CNVs. Rearrangements, while still rare, were more frequently observed in *PTEN* versus other genes (Fig. 1C, D). For all genes, CNVs were less frequently detected in the liquid versus the tissue biopsy cohort (Fig. 1C, D).

In assessing the liquid biopsy cohort with ctDNA TF ≥ 1% versus the non-paired tissue biopsy cohort, prevalence of pathogenic alterations in *PIK3CA*, *AKT1*, and *PTEN* was significantly higher in the liquid biopsy cohort (37.4% versus 47.5%, 5.0% versus 5.8%, and 12.4% versus 13.7%, respectively; Fig S2A). Within the liquid biopsy cohort with ctDNA TF ≥ 1%, prevalence of SVs in *PIK3CA*, *AKT1*, and *PTEN* was significantly higher than that in the non-paired tissue biopsy cohort (36.4% versus 47.1%, 4.4% versus 5.6%, and 7.2% versus 11.5%, respectively; Fig S2B).

Prevalence of CAPItello-defined alterations was 44.9% in the tissue biopsy cohort versus 34.3% in the liquid biopsy cohort (*P* < 0.001; Fig. 1A). In both cohorts, approximately two-thirds of samples with CAPItello-defined alterations harbored mutations in *PIK3CA* only (Fig S3).

### Concordance between tissue- and blood-based CGP

**Fig. 2:**
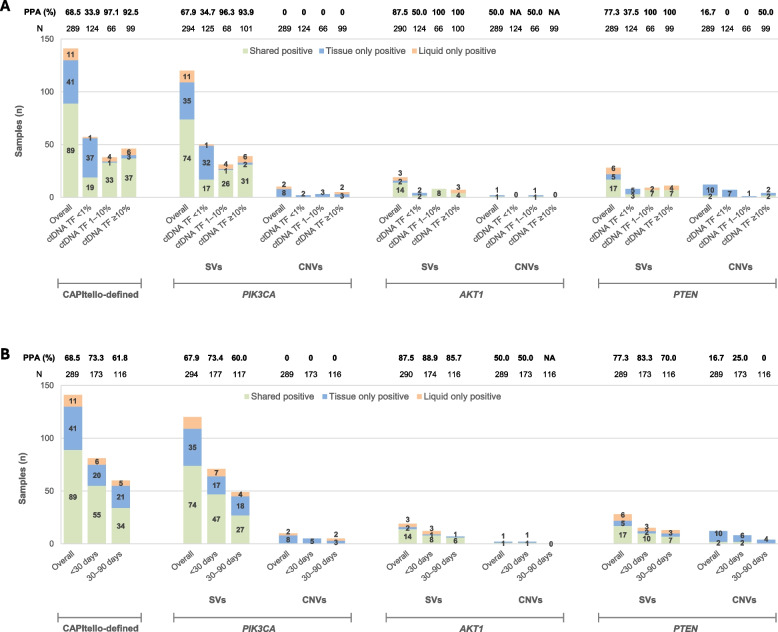
Positive predictive agreement in detection of pathogenic alterations between tissue and liquid biopsies according to (**A**) ctDNA TF and (**B**) collection intervals between paired biopsies. Please note that information on hormone receptor and human epidermal growth factor receptor 2 status (including hormone receptor-positive and triple-negative breast cancers) was not routinely available. Additionally, one sample may be counted in more than one category, e.g. both in ‘shared positive’ and ‘tissue-only positive’ for different alterations detected within this sample. Therefore, the total *N* shown here may exceed the number of samples (*n* = 289). PPA = shared positive / (shared positive + tissue-only positive)*. AKT1*, Akt serine/threonine kinase 1; CNV, copy number variant; ctDNA, circulating tumor DNA; NA, not applicable; *PIK3CA*, phosphatidylinositol-3-kinase catalytic subunit alpha; PPA, positive percent agreement; *PTEN*, phosphatase and tensin homolog; SV, short variant; TF, tumor fraction

In the 289 patients with paired CGP data, overall PPA was: *PIK3CA*, 67.9%; *AKT1*, 87.5%; *PTEN*, 77.3%; CAPItello-defined alterations, 68.5% (Fig. 2). PPA for all pathogenic *PIK3CA*, *AKT1*, and *PTEN* SVs, and for CAPItello-defined alterations, ranged from 92.5% to 100% for ctDNA TF ≥ 1% (Fig. 2A). CNVs were infrequent, and PPA varied across gene and ctDNA TF subgroups. For *PTEN* homozygous deletions none were detected in the ctDNA TF < 10% cases, and PPA was 50.0% (2/4) in cases with ctDNA TF ≥ 10% (Fig. 2A). All detected *AKT2* and *AKT3* alterations in the paired cohort comprised CNVs. Overall PPA for *AKT2* and *AKT3* CNVs was 66.7% (2/3; ctDNA TF ≥ 10% for all three samples) and 0% (0/12; ctDNA TF: < 1%, *n* = 4; 1%‍–10%, *n* = 2; ≥ 10%, *n* = 6), respectively. PPA was higher when paired biopsies were collected within < 30 versus 30–90 days of each other (Fig. 2B). The ctDNA TF breakdown within collection interval groups was as follows: < 30 days: < 1%, *n* = 68 (39.3%); 1%–10%, *n* = 35 (20.2%); ≥ 10%, *n* = 70 (40.5%); 30–90 days: < 1%, *n* = 56 (48.3%); 1%–10%, *n* = 31 (26.7%); ≥ 10%, *n* = 29 (25.0%). The percentage of samples with ctDNA TF < 1% did not differ significantly between groups (*P* = 0.146 by Fisher’s exact test). Overall NPA was high across all genes, ranging from 93.1% to 100% (Fig S4). Of note, 11 CAPItello-defined alterations were detected exclusively in liquid biopsies (*PIK3CA*, *n* = 8; *AKT1*, *n* = 1; *PTEN*, *n* = 2). On the other hand, 41 CAPItello-defined alterations were detected exclusively in tissue biopsies (*PIK3CA*, *n* = 25; *AKT1*, *n* = 1; *PTEN*, *n* = 9; multiple genes, *n* = 6), with the majority detected in tissue biopsies whose paired liquid biopsies had ctDNA TF < 1% (ctDNA ≥ 10%, *n* = 3; ctDNA TF 1%–10%, *n* = 1; ctDNA TF < 1%, *n* = 37).

### Co-occurring pathogenic alterations in the liquid biopsy cohort

**Fig. 3:**
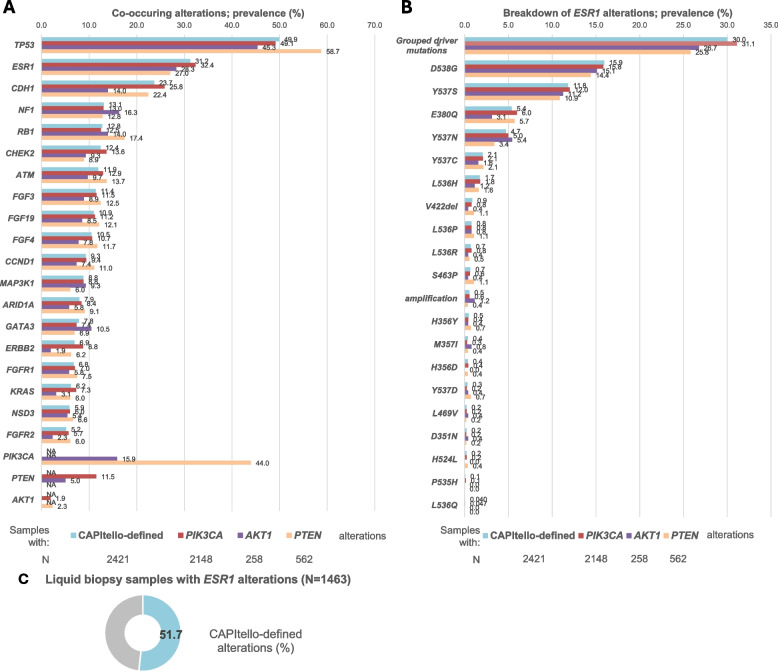
Co-occurring alterations in liquid biopsies. **A** Prevalence of co-occurring alterations in other clinically relevant genes. Prevalence is presented for pathogenic alterations in *PIK3CA*, *AKT1,* and *PTEN*, and in genes for which the prevalence of mutations was ≥ 5% in the cohort with CAPItello-defined alterations. Mutations in *DNMT3A*, *TET2*, and *ASXL1* are not presented, as they are likely associated with clonal hematopoiesis. **B** Breakdown of *ESR1* alterations in samples with CAPItello-defined alterations, and any pathogenic alterations in *PIK3CA*, *AKT1*, and *PTEN*. Prevalence is presented for alterations detected in ≥ 5 samples in the cohort with CAPItello-defined alterations and for individual grouped driver mutations irrespective of positive sample count. **C** Prevalance of co-occurring CAPItello-defined alterations in liquid biopsies with *ESR1* alterations. Please note that information on hormone receptor and human epidermal growth factor receptor 2 status (including hormone receptor-positive and triple-negative breast cancers) was not routinely available. ^a^Grouped driver mutations include the following: E380Q, S463P, P535H, L536R, L536Q, L536H, L536P, Y537S, Y537N, Y537C, and D538G. *AKT1*, Akt serine/threonine kinase 1; CNV, copy number variant; *ESR1*, estrogen receptor 1; NA, not applicable; *PIK3CA*, phosphatidylinositol-3-kinase catalytic subunit alpha; *PTEN*, phosphatase and tensin homolog; SV, short variant

In the overall cohort of liquid biopsies with CAPItello-defined alterations (*n* = 2,421), frequently co-occurring pathogenic alterations were observed in *TP53* (49.9%), *ESR1* (31.2%), *CDH1* (23.7%), *NF1* (13.0%), *RB1* (12.8%), *CHEK2* (12.4%), *ATM* (11.9%), *FGF3* (11.4%), *FGF19* (10.9%), and *FGF4* (10.5%), among others (Fig. 3A). Alterations in *BRCA1* (2.4%), *BRCA2* (4.8%), and *PALB2* (1.3%) were also observed. Similar distribution patterns were observed when pathogenic alterations in *PIK3CA*, *AKT1*, and *PTEN* were individually analyzed (Fig. 3A). In the breast cancer liquid biopsies that were positive for CAPItello-defined *PIK3CA*, *AKT1*, or *PTEN* alterations, the most frequently observed *ESR1* alterations (> 10.0%) were D538G and Y537S (Fig. 3B). The combined prevalence of *ESR1* driver mutations (E380Q, S463P, P535H, L536R, L536Q, L536H, L536P, Y537S, Y537N, Y537C, and D538G) in liquid biopsies with CAPItello-defined alterations was 30.0%. In the total liquid biopsy cohort, *ESR1* alterations were observed in 1,463 biopsies, 756 (51.7%) of which were also positive for CAPItello-defined alterations (Fig. 3C). Although we did not examine concordance of results in the liquid versus the tissue biopsy cohort for alterations in genes other than *PIK3CA*, *PTEN*, *AKT1*, *AKT2*, and *AKT3*, our results support the value of blood-based NGS panel testing in providing a comprehensive tumor mutational landscape.

## Discussion

In this study, we sought to compare the concordance of pathogenic alterations in *PIK3CA*, *AKT1*, *AKT2*, *AKT3*, and *PTEN* detected by tissue-based FoundationOne®CDx and blood-based FoundationOne®Liquid CDx in contemporaneous samples collected from patients with breast cancer. We assessed all alterations within or involving *PIK3CA*, *AKT1*, *AKT2*, *AKT3*, and *PTEN* that had the potential to activate the PI3K/AKT pathway. PPA between patient-paired liquid and tissue biopsy CGP data for the detection of CAPItello-defined alterations and SVs in *PIK3CA*, *AKT1*, and *PTEN* was high in cases with ctDNA TF ≥ 1%, suggesting that liquid biopsies with sufficient ctDNA shed may offer a less-invasive approach to accurately detect these alterations.

Efficiency of liquid biopsy NGS is influenced by tumor DNA shedding, which might not be as high in breast cancer as in other cancers such as small cell lung cancer and liver cancer [27]. Our results are in agreement with previous studies demonstrating that increased ctDNA TF improves concordance in the detection of tumor alterations between liquid and tissue biopsies [26, 27, 30–33]. It should be noted that samples with ctDNA TF < 1% were frequent in our study (52.6% of the total liquid biopsy cohort; 42.9% of the paired cohort) and in previous studies of patients with breast cancer (26.3%–31.2%) [27, 32]. PPA was higher in paired samples collected within 30 days versus 30–90 days of each other, as previously noted for shorter versus longer collection intervals [34–36]. Of note, ctDNA TF breakdown was similar between the two collection interval groups; therefore, differences cannot be attributed to lower shedding in the 30–90 days collection group. Differences were, nevertheless, minimal, as the interval was limited to 90 days to allow for comparisons at a similar point in the disease course. Although this may limit interpretation in the clinic, the main aim of this study was to assess the reliability of liquid vs tissue biopsy testing, rather than to uncover differences between archival samples and those collected at progression.

Detection of CNVs in ctDNA has been historically challenging, and ctDNA TF ≥ 10%–40% has been considered a requirement for reliable detection [21, 26, 37, 38]. In our study, we observed a 50.0% (2/4) PPA for *PTEN* homozygous deletions in samples with ctDNA TF ≥ 10%; results for CNVs across genes were generally variable. Of note, the FoundationOne®Liquid CDx assay is not approved for the detection of *PTEN* alterations. In the overall tissue and liquid biopsy cohorts, we observed a high proportion of CNVs among alterations detected in *AKT2*, *AKT3*, and *PTEN*, while rearrangements were more common in *PTEN* versus the other genes studied. Frequency of CNVs in the overall liquid versus tissue biopsies was lower, suggesting reduced sensitivity in the detection of CNVs in the liquid biopsy cohort, as expected [32]. Our results support the notion that reflex to tissue-based NGS in the case of a negative result with blood-based testing is important to enhance biomarker detection, especially for cases with ctDNA TF < 1% and for *PTEN* CNVs. These findings are reinforced by a recent study by Bhave et al. in patients with HR-positive/HER2-negative metastatic breast cancer, where *PTEN* homozygous deletion was the most common individual *PTEN* alteration detected by tissue-based CGP (4.1% of patients), but was detected in only four (0.2%) patients by blood-based CGP in non-paired samples [23]. Another recent study utilized a dataset of patients with advanced lung cancer and no actionable driver alterations found by blood-based NGS, who were subsequently tested with tissue-based NGS [33]. The study demonstrated that 52% (29/56) of the patients with ctDNA TF < 1% had a driver alteration identified by the subsequent tissue-based testing, providing evidence to support reflex to tissue biopsy in these cases [33]. In contrast, confirmatory tissue-based testing was negative for all patients (24/24) with ctDNA TF ≥ 1%, indicating that these patients may instead benefit from prompt treatment initiation [33].

Overall PPA for SVs in *PIK3CA*, *AKT1*, and *PTEN* between liquid and tissue biopsies in the paired cohort was 67.9%, 87.5%, and 77.3%, respectively. A meta-analysis of 25 studies previously reported a pooled PPA of 73% (95% CI, 70% to 77%) in the detection of *PIK3CA* mutations in liquid versus tissue biopsy [34]. Furthermore, in the plasmaMATCH trial, which investigated responses to capivasertib therapy, among others, in patients with advanced breast cancer, PPA for ctDNA digital polymerase chain reaction versus tissue-based NGS was 88.2% for *PIK3CA* (*n* = 77) and 88.9% for *AKT1* (*n* = 76) mutations defined as actionable [36]. In the LOTUS trial investigating the addition of the AKT inhibitor ipatasertib to paclitaxel in patients with metastatic TNBC, PPA for liquid versus tissue NGS was 100% for activating mutations in *PIK3CA* and *AKT1* (*n* = 89) [39]. *PTEN* alterations were not studied in these previous publications, due to limitations of the diagnostic methods at the time. Factors underlying PPA variability between the present and previous studies could include number of alterations studied, sample size, patient characteristics, differences in ctDNA TF measurement techniques, and inclusion of a combination of primary and metastatic tumor samples.

The higher prevalence of *PIK3CA*, *AKT1*, and *PTEN* alterations in the liquid biopsy cohort with ctDNA TF ≥ 1% versus the overall liquid biopsy cohort could be associated with improved sensitivity of detection in liquid biopsies with ctDNA TF ≥ 1%. Similarly, in Bhave et al., statistically significantly higher prevalence of *PIK3CA* mutations was noted in liquid biopsies with ctDNA TF ≥ 1% versus TF < 1%; prevalence of *AKT1* mutations and alterations involving *PTEN* was also numerically higher [23]. Regarding the differences between the liquid and tissue biopsy cohorts, alterations detected exclusively in liquid biopsies could be considered acquired alterations associated with tumor evolution and treatment resistance. However, polyclonality in liquid biopsies cannot be ruled out, as ctDNA is shed by all tumor sites versus the single-site sample collection for tissue biopsies; additionally, samples in the liquid and tissue biopsy cohorts were not paired.

Establishing concordance between tissue and liquid biopsy NGS testing is important as the number of targeted therapies and approved indications for liquid biopsy continues to expand. Our study is one of the largest analytical studies of pathogenic alterations in PI3K/AKT pathway genes (*PIK3CA, AKT1, AKT2, AKT3*, and *PTEN*) among patients with breast cancer. However, analysis of certain alterations, such as alterations in *AKT2* and *AKT3*, CNVs (including *PTEN* homozygous deletions), and gene rearrangements, was limited by low prevalence and/or technical limitations in blood-based CGP, e.g. insufficient ctDNA shed in some samples. Since HR/HER2 status was not available, analysis of CAPItello-defined alterations presented here should not be interpreted in the light of HR-positive/HER2-negative cancers. Biomarker-based treatment outcomes were also not available for this study, but future research may provide insights into prioritization of co-mutations when selecting second-line targeted therapy.

## Conclusions

We provide evidence that genomic profiling of ctDNA offers a minimally invasive potential alternative to identify clinically relevant *PIK3CA*/*AKT1*/*PTEN* SVs in cases with sufficient ctDNA shedding, i.e. ctDNA TF ≥ 1%. Analysis for *AKT2* and *AKT3* alterations was limited; however, for cases with negative liquid biopsy results, confirmation using tissue biopsy NGS should be considered to enhance detection of CNVs in general, and SVs when ctDNA TF is < 1%.

## Supplementary Information

Supplementary Material 1.

## Data Availability

The sequencing data generated in this study are derived from clinical samples. The data supporting the findings of this study are provided within the paper and its Data Supplement. All supplementary information accompanying the different analyses and figures presented in this study are provided in the Data Supplement. Due to HIPAA requirements, we are not consented to share individualized patient genomic data, which contains potentially identifying or sensitive patient information. Foundation Medicine is committed to collaborative data analysis, and we have well-established and widely utilized mechanisms by which investigators can query our core genomic database of > 700,000 deidentified sequenced cancers to obtain aggregated datasets. Requests for collaborative data shares can be made by contacting the corresponding author and filling out a data collaboration committee form. Once approved, investigators are required to sign a data transfer agreement. Written proposals are considered at quarterly meetings and data transfer agreements expire 18 months from execution of the agreement.

## Abbreviations

AKT: Akt serine/threonine kinase
CGP: Comprehensive genomic profiling
CNV: Copy number variant
ctDNA: Circulating tumor DNA
*ESR1*: Estrogen receptor 1
FDA: US Food and Drug Administration
FDR: False discovery rate
HER2: Human epidermal growth factor receptor 2
HR: Hormone receptor
PI3K: Phosphatidylinositol-3-kinase
PPA: Positive percentage agreement
NGS: Next-generation sequencing
NPA: Negative percentage agreement
SNV: Single nucleotide variant
SV: Short variant
TF: Tumor fraction
VAF: Variant allele frequency
