# Supplementary material for: Retrospective comparison between breast cancer tissue- and blood-based next-generation sequencing results in detection of PIK3CA, AKT1, and PTEN alterations
Source: Breast Cancer Res. 2025 Jul 1;27:122. doi: 10.1186/s13058-025-02055-0 (PMC12217283; doi:10.1186/s13058-025-02055-0)
Supplement: Supplementary file 1 — Supplementary Material 1. [file 13058_2025_2055_MOESM1_ESM.pdf]

DATA SUPPLEMENT

Retrospective Comparison Between Breast Cancer Tissue- and Blood-Based Next-Generation Sequencing Results in Detection of *PIK3CA*, *AKT1*, and *PTEN* Alterations

Moumita Chaki, PhD[1]; Mona Benrashid, PharmD, BCOP[1]; Subir Puri, PharmD[1]; Smruthy Sivakumar, PhD[2]; Ethan S. Sokol, PhD[2]; Josefa M. Briceno, MD[1]; and Neil Vasan, MD, PhD[3]

[1]Breast Cancer, US Medical Affairs, AstraZeneca, Gaithersburg, MD

[2]Computational Discovery, Foundation Medicine Inc, Cambridge, MA

[3]Columbia University Irving Medical Center, New York, NY

CORRESPONDING AUTHOR

Moumita Chaki, PhD; e-mail: moumita.chaki@astrazeneca.com

TABLE S1. List of Alterations Defined in the CAPItello-291 Protocol

**Gene**	**Alteration**
AKT1	SVs<br><br>E17K
PIK3CA	SVs<br><br>R88Q<br><br>E545A<br><br>Q546E<br><br>M1043V<br><br>H1047Y<br><br>G1049R<br><br>N345K<br><br>E545D<br><br>Q546K<br><br>M1043I<br><br>H1047R<br><br>C420R<br><br>E545Q

	Q546R<br><br>H1047L<br><br>E542K<br><br>E545K<br><br>E545G<br><br>Q546P
PTEN	SVs<br><br>C124R<br><br>G129E<br><br>R130Q<br><br>C136R<br><br>S170R<br><br>R173C<br><br>C124S<br><br>G129V<br><br>R130G<br><br>C136Y<br><br>G129R

	R130L<br><br>R130P<br><br>Any nonsense, frameshift, or splice site alteration (missense mutations in the start codon and SV deletions spanning from upstream of the start codon are included in this category)
	Homozygous copy number deletion
	Predicted pathogenic rearrangement in *PTEN*

AKT1, Akt serine/threonine kinase 1; *PIK3CA*, phosphatidylinositol-3-kinase catalytic subunit alpha; *PTEN*, phosphatase and tensin homolog; SV, short variant.

TABLE S2. Patient Characteristics in the Liquid Biopsy Cohort With ctDNA TF ≥1%

**Characteristic**	**Liquid biopsy cohort with ctDNA TF ≥1% (*N* = 3,344)**
Age (years), median (interquartile range)	63 (55-72)
Sex, n (%)	
Male	38 (1.1)
Female	3,306 (98.9)
Tumor type, n (%)	
Breast/breast carcinoma (not otherwise specified)	2,664 (79.7)
Breast invasive ductal carcinoma	533 (15.9)
Breast invasive lobular carcinoma	141 (4.2)
Breast metaplastic carcinoma	4 (0.1)
Other breast[a]	2 (0.1)
Tumor stage, n (%)	
I	67 (2.0)
II	117 (3.5)
III	129 (3.9)
IV	2,358 (70.5)
Unknown	673 (20.1)

Please note that information on hormone receptor and human epidermal growth factor receptor 2 status (including hormone receptor-positive and triple negative breast cancers) was not routinely available. [a]Other breast: breast mucinous carcinoma, breast inflammatory carcinoma. ctDNA, circulating tumor DNA; TF, tumor fraction.

TABLE S3. Characteristics in Patients With Paired Tissue and Liquid Biopsy CGP Data

**Characteristic**	**Paired cohort (*N* = 289)**	
	**Tissue biopsy**	**Liquid biopsy**
Age (years), median (interquartile range)	61 (52-71)	61 (52-71)
Sex, n (%)		
Male	7 (2.4)	7 (2.4)
Female	282 (97.6)	282 (97.6)
Tumor type, n (%)		
Breast/breast carcinoma (not otherwise specified)	214 (74.0)	227 (78.5)
Breast invasive ductal carcinoma	61 (21.1)	50 (17.3)
Breast invasive lobular carcinoma	13 (4.5)	12 (4.2)
Breast metaplastic carcinoma	1 (0.3)	0
Other breast[a]	0	0
Tumor stage, n (%)		
I	10 (3.5)	8 (2.8)
II	16 (5.5)	19 (6.6)
III	18 (6.2)	22 (7.6)
IV	188 (65.1)	181 (62.6)
Unknown	57 (19.7)	59 (20.4)

Please note that characteristics might differ for each patient at tissue versus liquid biopsy collection, given these occurred at different timepoints. Additionally, information on hormone receptor and human epidermal growth factor receptor 2 status (including hormone receptor-positive and triple negative breast cancers) was not routinely available. [a]Other breast: breast mucinous carcinoma, breast inflammatory carcinoma. CGP, comprehensive genomic profiling.

FIG S1. Study analysis sets. *AKT1*, Akt serine/threonine kinase 1; CGP, comprehensive genomic profiling; ctDNA, circulating tumor DNA; *ESR1*, estrogen receptor 1; *PIK3CA*, phosphatidylinositol-3-kinase catalytic subunit alpha; *PTEN*, phosphatase and tensin homolog; TF, tumor fraction.

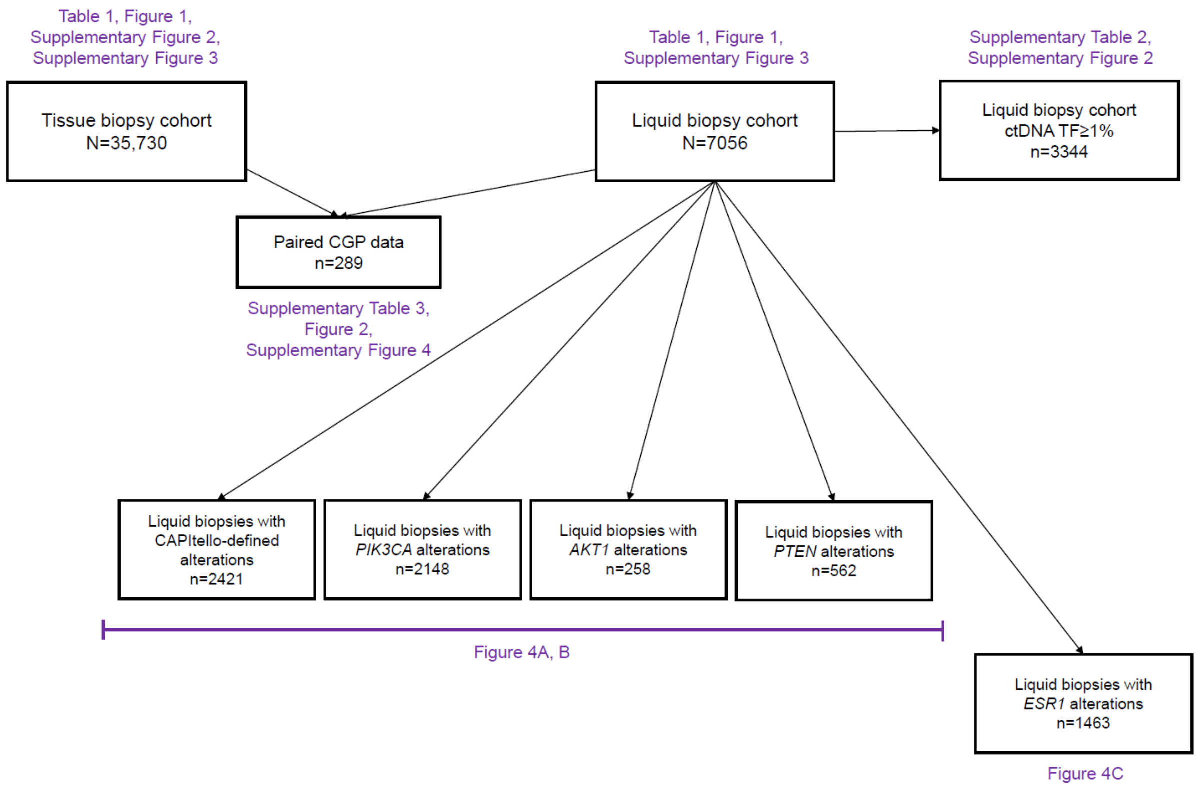

FIG S2. Prevalence of (A) all pathogenic alterations and (B) pathogenic short variants in the tissue and liquid biopsy ctDNA TF ≥1% cohorts. *P < .05; ** P < .01; *** P < .001. Please note that information on hormone receptor and human epidermal growth factor receptor 2 status (including hormone receptor-positive and triple negative breast cancers) was not routinely available. *AKT1*, Akt serine/threonine kinase 1; *AKT2*, Akt serine/threonine kinase 2; *AKT3*, Akt serine/threonine kinase 3; ctDNA, circulating tumor DNA; *PIK3CA*, phosphatidylinositol-3-kinase catalytic subunit alpha; *PTEN*, phosphatase and tensin homolog; TF, tumor fraction.

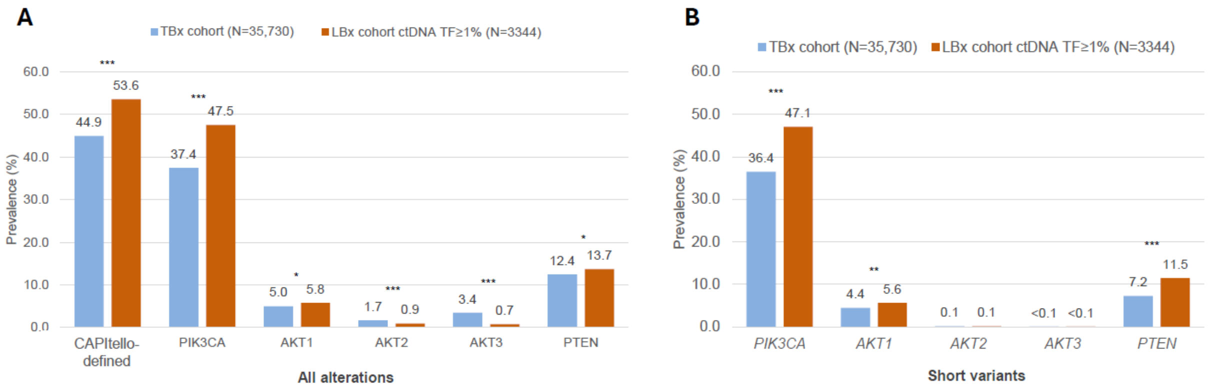

FIG S3. Breakdown of *PIK3CA*, *AKT1*, and *PTEN* pathogenic alterations[a] in breast cancer samples with CAPItello-defined alterations. Please note that information on hormone receptor and human epidermal growth factor receptor 2 status (including hormone receptor-positive and triple negative breast cancers) was not routinely available. [a]*PIK3CA*, *PTEN*, and *AKT1* alterations defined in the CAPItello-291 trial protocol (Table S1). *AKT1*, Akt serine/threonine kinase 1; *PIK3CA*, phosphatidylinositol-3-kinase catalytic subunit alpha; *PTEN*, phosphatase and tensin homolog. **FIG S4.** Negative predictive agreement in detection of alterations between tissue and liquid biopsies according to (A) ctDNA TFs and (B) collection intervals between paired biopsies. Please note that information on hormone receptor and human epidermal growth factor receptor 2 status (including hormone receptor-positive and triple-negative breast cancers) was not routinely available. Additionally, one sample may be counted in more than one category, e.g. both in 'shared positive' and 'tissue-only positive' for different alterations detected within this sample. Therefore, the total *N* shown here may exceed the number of samples (n = 289). NPA = shared negative / (shared negative + liquid-only positive). *AKT1*, Akt serine/threonine kinase 1; *AKT2*, Akt serine/threonine kinase 2; *AKT3*, Akt serine/threonine kinase 3; CNV, copy number variant; ctDNA, circulating tumor DNA; NPA, negative percent agreement; *PIK3CA*, phosphatidylinositol-3-kinase catalytic subunit alpha; *PTEN*, phosphatase and tensin homolog; SV, short variant; TF, tumor fraction.

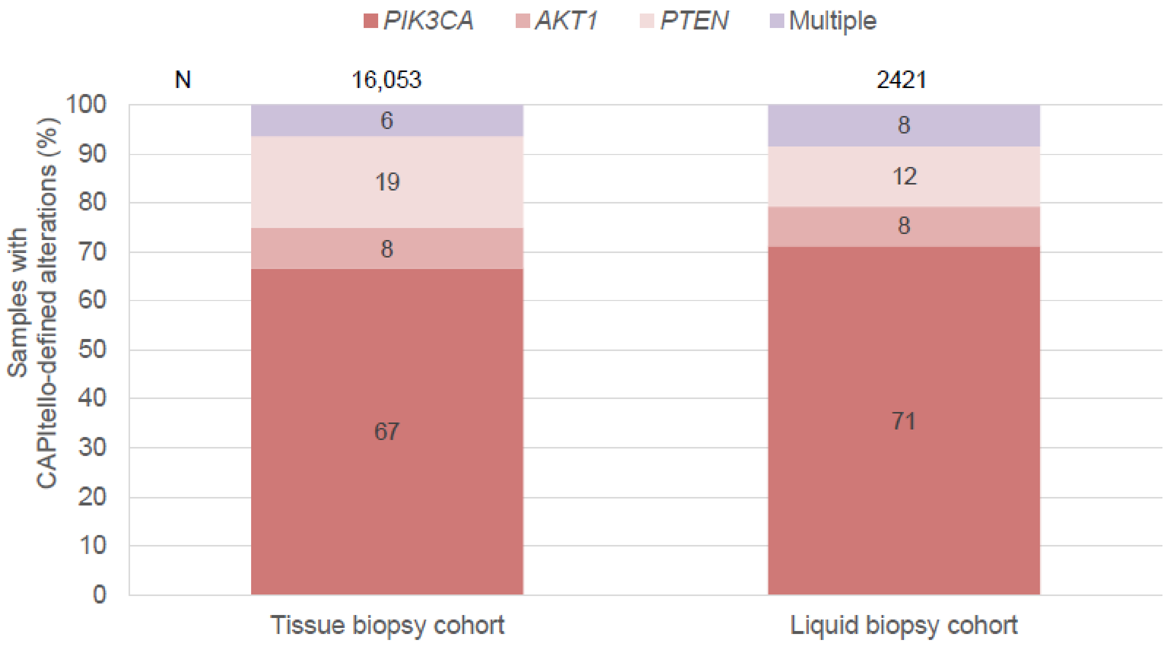

FIG S4. Negative predictive agreement in detection of alterations between tissue and liquid biopsies according to (A) ctDNA TFs and (B) collection intervals between paired biopsies. Please note that information on hormone receptor and human epidermal growth factor receptor 2 status (including hormone receptor-positive and triple-negative breast cancers) was not routinely available. Additionally, one sample may be counted in more than one category, e.g. both in 'shared positive' and 'tissue-only positive' for different alterations detected within this sample. Therefore, the total *N* shown here may exceed the number of samples (*n* = 289). NPA = shared negative / (shared negative + liquid-only positive). *AKT1*, Akt serine/threonine kinase 1; *AKT2*, Akt serine/threonine kinase 2; *AKT3*, Akt serine/threonine kinase 3; CNV, copy number variant; ctDNA, circulating tumor DNA; NPA, negative percent agreement; *PIK3CA*, phosphatidylinositol-3-kinase catalytic subunit alpha; *PTEN*, phosphatase and tensin homolog; SV, short variant; TF, tumor fraction.

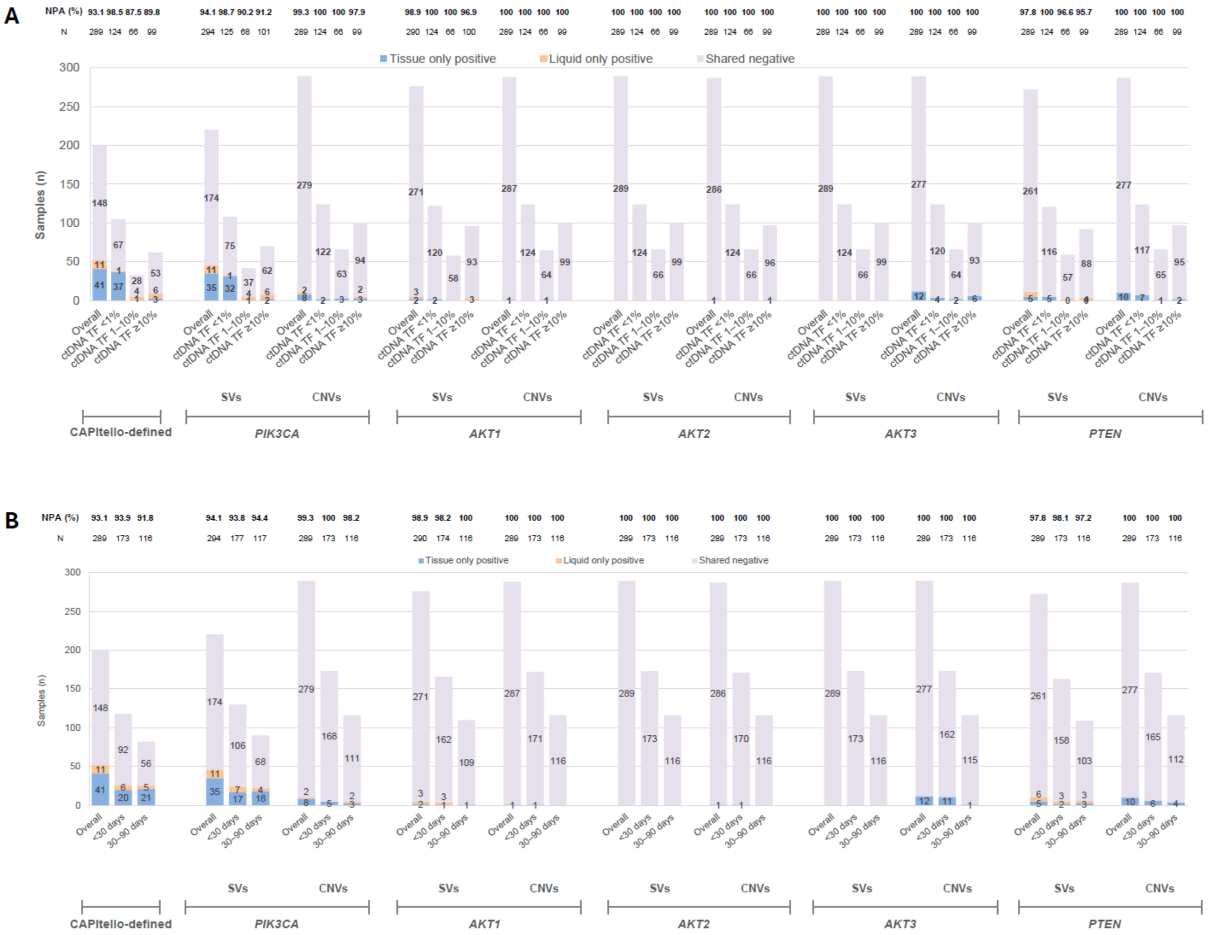

A
B
NPA (%)
N
Tissue only positive
Liquid only positive
Shared negative
Samples (n)
SVs
CNVs
CAPItello-defined
PIK3CA
AKT1
AKT2
AKT3
PTEN
Overall
ctDNA TF <1%
ctDNA TF 1–10%
ctDNA TF ≥10%
<30 days
30–90 days
